# Microstructural Predictors of Cognitive Impairment in Cerebral Small Vessel Disease and the Conditions of Their Formation

**DOI:** 10.3390/diagnostics10090720

**Published:** 2020-09-19

**Authors:** Larisa A. Dobrynina, Zukhra Sh. Gadzhieva, Kamila V. Shamtieva, Elena I. Kremneva, Bulat M. Akhmetzyanov, Ludmila A. Kalashnikova, Marina V. Krotenkova

**Affiliations:** Research Center of Neurology, 80 Volokolamskoe shosse, 125367 Moscow, Russia; kamila.shamt@gmail.com (K.V.S.); moomin10j@mail.ru (E.I.K.); doctorbulat@mail.ru (B.M.A.); kalashnikovancn@yandex.ru (L.A.K.); krotenkova_mrt@mail.ru (M.V.K.)

**Keywords:** cerebral small vessel disease, diffusion tensor imaging, phase-contrast magnetic resonance imaging, cerebrospinal fluid and blood flow, cognitive impairment

## Abstract

Introduction: Cerebral small vessel disease (CSVD) is the leading cause of vascular and mixed degenerative cognitive impairment (CI). The variability in the rate of progression of CSVD justifies the search for sensitive predictors of CI. Materials: A total of 74 patients (48 women, average age 60.6 ± 6.9 years) with CSVD and CI of varying severity were examined using 3T MRI. The results of diffusion tensor imaging with a region of interest (ROI) analysis were used to construct a predictive model of CI using binary logistic regression, while phase-contrast magnetic resonance imaging and voxel-based morphometry were used to clarify the conditions for the formation of CI predictors. Results: According to the constructed model, the predictors of CI are axial diffusivity (AD) of the posterior frontal periventricular normal-appearing white matter (pvNAWM), right middle cingulum bundle (CB), and mid-posterior corpus callosum (CC). These predictors showed a significant correlation with the volume of white matter hyperintensity; arterial and venous blood flow, pulsatility index, and aqueduct cerebrospinal fluid (CSF) flow; and surface area of the aqueduct, volume of the lateral ventricles and CSF, and gray matter volume. Conclusion: Disturbances in the AD of pvNAWM, CB, and CC, associated with axonal damage, are a predominant factor in the development of CI in CSVD. The relationship between AD predictors and both blood flow and CSF flow indicates a disturbance in their relationship, while their location near the floor of the lateral ventricle and their link with indicators of internal atrophy, CSF volume, and aqueduct CSF flow suggest the importance of transependymal CSF transudation when these regions are damaged.

## 1. Introduction

Cerebral small vessel disease (CSVD)/cerebral microangiopathy, associated with age and vascular risk factors, is the main cause of vascular and mixed degenerative cognitive impairment (CI) [1,2,3,4,5,6]. According to the neuropathological data, concomitant vascular and degenerative brain damage increases the risk of dementia by more than twofold, thus representing the dominant cause of CI in the elderly [2,3,5]. MRI is the main method for diagnosing CSVD and clarifying the effects of this pathology on the development of degenerative lesions [7,8,9]. Among the MRI signs of CSVD, progressive white matter lesions (T2/FLAIR white matter hyperintensities (WMH)/leukoaraiosis) are the most closely associated with cognitive decline [10]. Diffusion tensor imaging (DTI) is especially significant in the study of CI due to CSVD [6,11,12,13,14]. DTI, which uses water diffusion to quantitatively measure the state of the brain’s white matter microstructure, has shown significant advantages in characterizing CSVD severity during the evaluation of normal-appearing white matter (NAWH) [11,12,14]. The availability of sensitive DTI predictors of CI in CSVD is very important for clinical trials since the slow rate of CI requires treatment efficacy to be evaluated in a very large sample [15,16]. In mild cognitive impairment (MCI) patients, the mean diffusivity (MD) and fractional anisotropy (FA) were found to be correlated with the Montreal Cognitive Assessment (MoCA), a screening tool used for vascular cognitive impairment [17]. The reduced FA and increased MD in NAWH were found to be better compared to the severe WMH, lacunae, and atrophy in the evaluation of the neuropsychological changes characteristic of CSVD, such as decreased executive function and information processing speed [18,19,20,21,22]. The DTI markers of demyelination, radial diffusivity (RD), and axonal degeneration (axial diffusivity (AD)) [23,24] were characterized by an increase in NAWH in CSVD with moderate CI [25]. RD offers better predictability of a reduction in executive function than AD [25]. Williams et al. (2017, 2019) proposed a method for automatic DTI segmentation to obtain the coefficient of a surrogate measure of CSVD severity associated with executive dysfunction, the speed of information processing, global cognition, and predictive CI over several years of observation [26,27]. At the same time, the use of DTI methods that consider the distribution of changes reveals regional damage selectivity, which corresponds to impairments of specific cognitive functions [20,28,29] and allows the differentiation between vascular and degenerative dementia due to Alzheimer’s disease [30]. Different parts of the corpus callosum are commonly indicated as regions where CI and dementia type can be differentiated [20,29,30]. Thus, a region of interest (ROI) analysis, based on the diffusion metrics associated with severity and predominantly demyelinating or axonal damage [23], may provide better data on the conditions of CI development due to the complex neuropsychological profile of CSVD [25,31,32,33], as a consequence of the heterogeneity of the damage mechanisms and high comorbidity with degeneration in CI progression [12,14]. Whether deep or periventricular white matter damage is the leading cause of CI remains unsolved. Post-mortem imaging and neuropathological studies suggest that changes in the periventricular and deep white matter have different etiologies [14], which are confirmed by differences in the neuropathological predictors of periventricular and deep WMH [34]. In the present study, different regions of WMH in the hemispheres, corpus callosum, cingulate gyri, and hippocampi were selected as the ROIs, which were evaluated using FA, MD, AD, and RD metrics in patients with CSVD and CI of varying severity. These data were used to construct a predictive model of CI that can reflect the leading conditions for its development in patients with MRI signs of CSVD. The association between DTI predictors and blood flow, cerebrospinal fluid flow, WMH, and atrophy was thus clarified.

## 2. Materials and Methods

### Study Population

This study included 74 patients aged 46–70 years who presented with cognitive complaints (difficulties with concentration, memory and clear thinking) and whose brain changes on MRI corresponded to CSVD (lacunae, WMH, enlarged perivascular spaces, microbleeds, and cerebral atrophy) [7]. Patients with Fazekas grade 1 WMH were included in the study if they had arterial hypertension (AH) stage 2 or 3 [35] and/or ≥1 lacunae. 

The exclusion criteria were as follows: (1) severe dementia [36,37]; (2) CI caused by probable Alzheimer’s disease according to the National Institute on Aging (United States) criteria [38,39]; (3) patients with small subcortical infarcts/lacunae < 3 months after an acute cerebrovascular event; (4) CSVD caused by other independent causes (genetic, inflammatory, thrombophilic, systemic or toxic causes, or a history of severe migraines); (5) a different cause of stroke and a concomitant brain pathology other than CSVD; (6) > 50% atherosclerotic stenosis of the extra- or intracranial arteries; (7) a serious health condition, such as a cardiac disorder (ejection fraction < 50%), an endocrine condition (diabetes mellitus (DM) type 1 or 2 with severe vascular complications), an uncompensated thyroid disorder, or renal failure (chronic kidney disease with a glomerular filtration rate < 30 mL/min); (8) uncontrolled AH [40]; or (9) contraindications from MRI studies.

These patients were selected from a total group of 96 patients with CSVD, examined at the Research Center of Neurology (Moscow, Russia) between January 2016 and December 2017 [41], based on a complete MRI protocol, including DTI and phase contrast studies.

The control group consisted of 18 volunteers (12 women; average age 57.8 ± 5.9 years) with no clinical or MRI evidence of vascular or degenerative brain pathology who were matched for age and gender. 

This study was approved by the Local Ethics Committee of the Research Centre of Neurology (Moscow, Russia). The ethics statement number is 1-8/16, dated 27 January 2016. All subjects signed an informed consent form for participation in the study and for processing of their personal data.

The presence of classic vascular risk factors (AH [35], hypercholesterolemia [42], obesity [43], DM type 2, and smoking) was assessed in the patients and controls.

CI severity was measured using the MoCA [36] and independence in daily life [37]: A score of >26 points and cognitive complaints were considered subjective cognitive impairment (SCI), <26 points and independence were considered MCI, and <26 points and dependence were considered dementia. All study participants were right-handed.

## 3. MRI Protocol and Imaging Analysis

### 3.1. Routine MRI

MRI data were acquired using a Siemens MAGNETOM Verio 3T scanner (Siemens Medical Systems, Erlangen, Germany) with a standard 12-channel matrix head coil. To evaluate the STRIVE criteria [7], the patients and control group underwent axial spin echo T2-weighed imaging (TR 4000 ms; TE 118 ms; slice thickness 5.0 mm; plane resolution, 1.5 mm^2^; duration: 2 min 02 s; 22 slices); sagittal 3D T2 FLAIR imaging (TR 6000 ms; TE 395 ms; isotropic voxel 1 × 1 × 1 mm; duration: 7 min 12 s; 160 slices); sagittal 3D T1-mpr imaging (TR 1900 ms; TE 2.5 ms; isotropic voxel 1 × 1 × 1 mm; duration: 4 min 16 s; 176 slices); diffusion MRI (DWI) using an axial spin-echo echo-planar imaging sequence with two b-values (0, 1000 s/mm2) (TR—4000 ms, TE—100 ms, slice thickness—4 mm, duration: 1 min 20 s; 25 slices); and an axial susceptibility weighted imaging sequence (SWI) with magnitude and phase image reconstruction (TR 28 ms; TE 20 ms; slice thickness 1.2 mm; FOV 179 × 230 mm, duration: 8 min 12 s; 88 slices). Two neuroradiologists, blinded to clinical information, evaluated the brain MR imaging studies in a standardized manner. No STRIVE criteria were found in the control group. For the patient group, there were no acute or recent small lacunar infarcts based on the DWI analysis. The Fazekas scale was used to quantify T2 FLAIR WMH (grade 0–3) [44]. 

### 3.2. Diffusion-Tensor Imaging

The DTI examination was performed using a productive spin-echo echo-planar imaging sequence with three *b*-values (0, 1000, and 2500 s/mm^2^) and 64 non-coplanar diffusion directions for each non-zero *b*-value (TE/TR 115/12600 ms; matrix 100 × 100, resolution 2 × 2 × 2 mm^3^; 64 slices; the number of excitations was equal 1; the GRAPPA acceleration factor was 2) [45]. DTI was used to build FA, MD, RD, and AD maps followed by a regions of interest (ROIs) analysis. ROI 25,87 mm^3^ in size (4 voxels in the axial plane) were manually selected by two neuroradiologists (K.E.I., A.B.M., with 10 years of work experience) independently of each other on b0 images, followed by projection on 4 diffusion metric maps using the ITK-SNAP software (http://itksnap.org). The reliability of the selected DTI values of each ROI was evaluated using intraclass coefficients, which were > 0.7 in all cases. The subsequent analysis used an average volume of two ROI measurements. 

**Figure 1:**
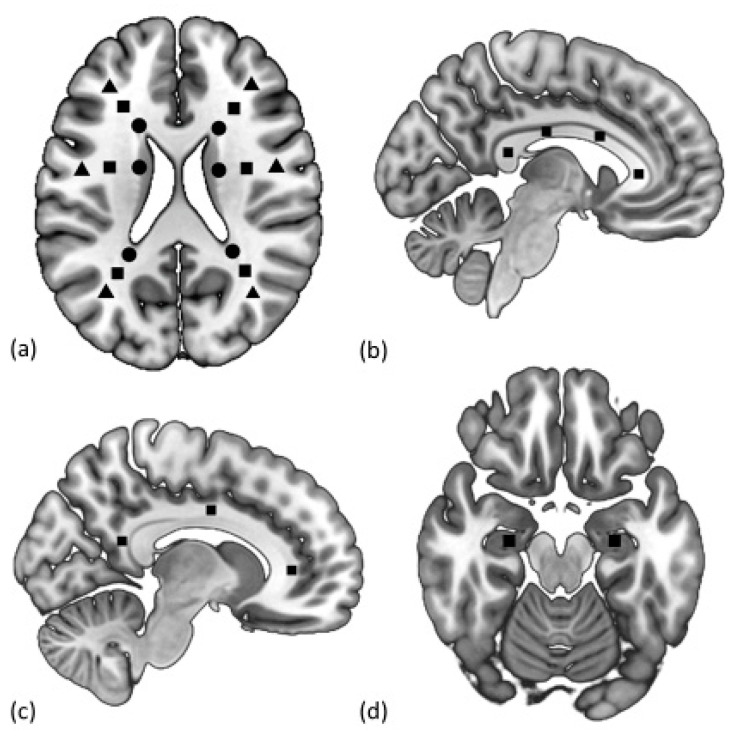
Selection of the regions of interest (ROIs) in the periventricular (circle), deep (square), and juxtacortical (triangle) normal-appearing white matter (NAWM) (**a**); anterior, mid-anterior, mid-posterior, and posterior corpus callosum (CC) (**b**); anterior, middle, and posterior cingulum bundle (CB) (**c**); and hippocampi (**d**).

**Figure A1:**
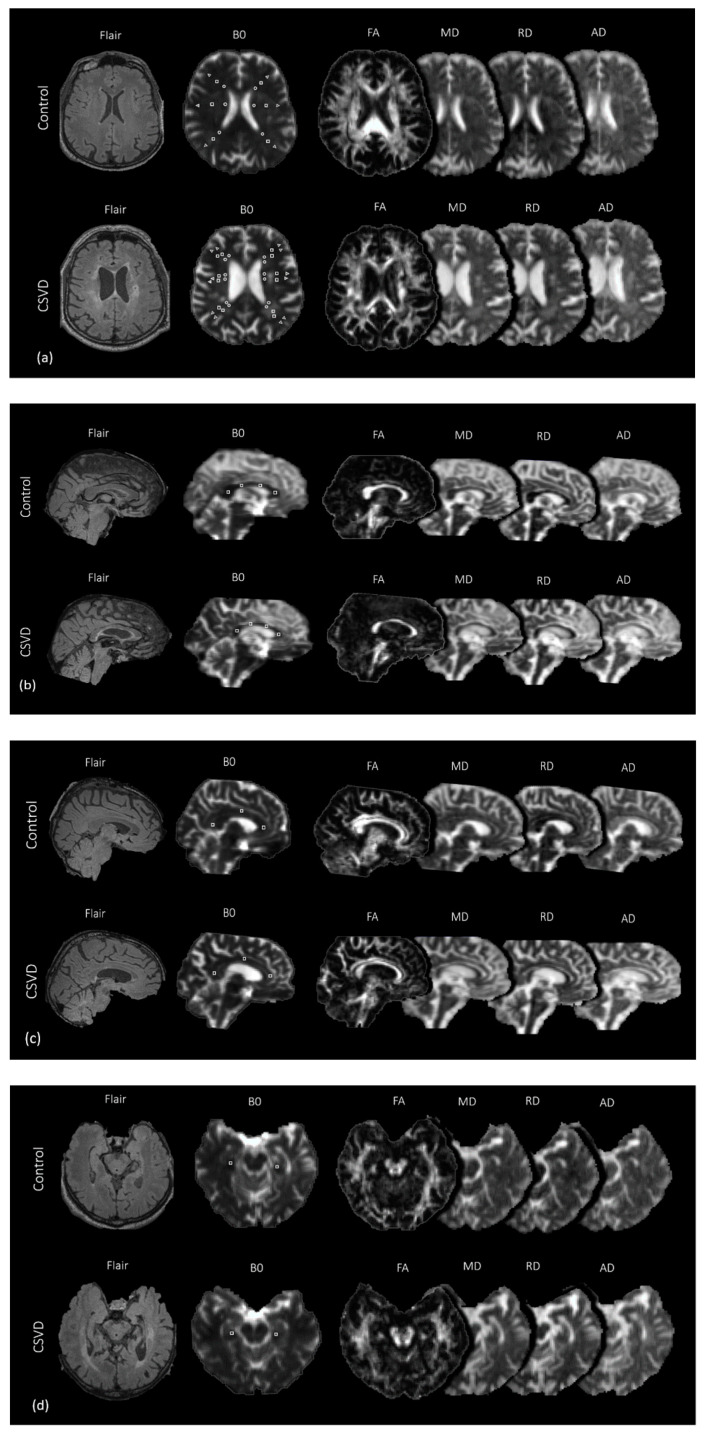
Selection of ROI in the periventricular (circle), deep (square), and juxtacortical (triangle) WMH and NAWM (**a**); anterior, mid-anterior, mid-posterior, and posterior CC (**b**); anterior, middle, and posterior CB (**c**); and hippocampi (**d**) on b0 images, followed by projection on 4 diffusion metric maps (FA, MD, RD, AD).

The selected ROIs are shown in Figure 1 below, and the MRI images are presented in Figure A1 of the Appendix A. These ROIs included the centers of the anterior, anteromedial, posteromedial, and posterior sections of the corpus callosum [46]; the anterior, middle, and posterior parts of the cingulate gyri [47]; hippocampal heads; and normal-appearing white matter (NAWM) of the hemispheres [48]. NAWM ROIs were painted on a slice at the level of the bodies of the lateral ventricles, above the subcortical structures. Then, the ROIs were highlighted on the axes of the anterior (anterofrontal region) and posterior (parietotemporal region) horns of the lateral ventricles, the body of the lateral ventricle (posterofrontal region) on the axial images, separately for periventricular (3–13 mm from the wall of the lateral ventricle), juxtacortical (4 mm from the corticomedullary junction), and deep (between the two described zones) white matter. ROI selection control was achieved by checking the locations of the markers in all three projections (axial, sagittal, and frontal) using a 3D cursor, while NAWM was verified using the location of the WMH on the T2/FLAIR images. In the absence of NAWM in the ROI projection, the ROI was selected vertically (1–2 slices above and below) and horizontally (laterally up to 5 mm from the main axis). The obtained zones were preserved as binary masks and were used to further assess the diffusion metrics values in each area.

All ROIs of the corpus callosum (CC), cingulum bundle (CB), and NAWM of the left hemisphere were included in the predictive model. The latter had more significant deviations in CSVD from the average values of the control for a larger number of zones compared to the ROIs in the right hemisphere, in the absence of differences between the ROIs of the NAWM of the left and right hemispheres. This choice, in particular, was justified by the dominance of the left hemisphere in all the studied patients. 

### 3.3. Phase Contrast MRI

Cardiac-gated phase contrast MRI (PC–MRI) was used to estimate the blood flow, cerebrospinal fluid flow (CSF flow), and aqueduct surface area (sAq). The cardiac cycle was covered in 32 frames. The scanning parameters were TR = 28.7 ms, TE = 8 ms, slice thickness 5.0 mm, field of view 101 × 135 mm, matrix 256 × 192 pixels, number of excitations (averages) = 1, velocity encoding value (Venc) for CSF flow from 5 to 20 cm/s, and for blood flow, from 60 to 80 cm/s. The slice plane was strictly perpendicular to the direction of blood flow in the internal carotid and vertebral arteries, the straight and superior sagittal sinuses, and in the direction of CSF flow at the level of the cerebral aqueduct. Images were processed using the Bio Flow Image Flow Analysis Software, Version 04.12.16, http://tidam.fr/, which facilitated vessel and aqueduct lumen segmentation and quantification of the blood flow and CSF flow. Calculations included the total arterial blood flow (tABF) in the internal carotid and vertebral arteries (ml/min); the superior sagittal sinus venous blood flow (sssVBF) (ml/min); the straight sinus venous blood flow (stVBF) (ml/min); the aqueduct cerebrospinal fluid flow (aqCSF flow) (mm^3^/sec); the pulsatility index (Pi) according to the formula Pi = (Vmax-Vmin)/Vmean, where Vmean is the mean arterial blood flow during the cardiac cycle, while Vmax and Vmin are the maximum and minimum values of arterial blood flow, respectively; and sAq (mm^2^), which was used to semi-automatically calculate images as the area of the aqueduct at the level of the midbrain (axial slice).

### 3.4. Brain Segmentation

Voxel-based morphometry was used to calculate the total brain volume (tBV) (cm3), total CSF (tCSF) (cm3), total white matter (tWM) (cm3), total gray matter (tGM) (cm3), and the volume of lateral ventricles (vLV) (cm3) [49]. The procedure for processing T1-weighted images with SPM 12 (http://www.fil.ion.ucl.ac.uk/spm/software/spm12) based on the MATLAB R2016a software (9.0.0.341360) included a preliminary coregistration of images with 3D-FLAIR images and segmentation of the structural images into gray matter, white matter, and cerebrospinal fluid, followed by correction of the gray and white matter images, taking into account the presence of WMH in the main group (WMH masks obtained during 3D-FLAIR image processing were used for this purpose). Then, the DARTEL algorithm was used to create a common template of gray and white matter of all examined subjects (the main and control groups), normalized in stereotactic MNI space (Montreal Neurological Institute template), followed by normalization and modulation for comparison of the volumes between groups and smoothing of individual gray and white matter files. The total volume of gray matter and CSF space was calculated using a script based on MATLAB R2016a (9.0.0.341360), using the utility get_totals by Ged Ridgway (http://www0.cs.ucl.ac.uk/staff/g.ridgway/vbm/get_totals.m). To calculate the volume of WMH (vWMH) (cm3), 3D-FLAIR images were reduced to a single stereotactic MNI space (Montreal Neurological Institute) in the SPM12 program (http://www.fil.ion.ucl.ac.uk/spm). Sequential segmentation of WMH was then performed in the LST program [50] with manual correction and volume calculation in the ITK-SNAP program (http://itksnap.org). The obtained data were saved as a binary mask and subsequently used to create the NAWM mask. 

### 3.5. Statistical Analysis

The statistical analysis was performed using the IBM SPSS 23.0 (IBM Corp, 2015; Armonk, NY, USA) and R 3.4.3 software (Core Tea, 2017; Vienna, Austria:). The main descriptive statistics for categorical and ordinal variables were the frequency and proportion (%); mean, standard deviation, minimum (min.), and maximum (max.) or median; and interquartile range for quantitative variables. Two-way versions of the statistical criteria were used in all cases. The null hypothesis was rejected if *p* < 0.05. The qualitative parameters for grouping variable levels were compared using the χ2 test or Fisher’s exact test. Quantitative parameter values were compared using the Student’s t-test or the Mann–Whitney *U* test depending on the type of their distribution. The normality was checked out using the Kolmogorov–Smirnov test and visually with histograms. In the case of multiple comparisons, the Benjamini–Hochberg method was used to reduce the number of false discoveries. Pearson’s correlation analysis was used to assess the relationship between the quantitative parameters. Binary logistic regression was used to assess the ability of individual parameters to predict the development of CI in patients with CSVD, where 0 was taken as a binary outcome for SCI and 1 indicated MCI and dementia. All obtained DTI data were included in the logistic regression, which subsequently created an equation featuring the most statistically significant predictor indicators. The selection of predictors was performed by the step-by-step method with the calculation of the coefficient of determination and subsequent assessment of the Hosmer–Lemeshow goodness-of-fit test. The efficacy of the chosen logistical model was further evaluated by a receiver operating characteristic (ROC) analysis according to the probabilities predicted by the model (binary outcome), including calculation of the area under the curve, the ideal cut-off points, and their sensitivity and specificity.

## 4. Results

### 4.1. Participant Characteristics

The characteristics of the study patients with cerebral small vessel disease (CSVD) and the controls are presented in Table 1. 

There were no differences in age, sex, education level, smoking, hypercholesterolemia, or obesity between groups. Hypertension was significantly more common in patients with CSVD. The range of CI was as follows: dementia in 12 (16.2%) patients, MCI in 33 (44.6%) patients, and SCI in 29 (39.2%) patients. The MRI signs of CSVD were as follows: Fazekas grade 1 WMH in 19 (25.6%) subjects, Fazekas grade 2 in 23 (31.1%) subjects, Fazekas grade 3 in 32 (43.3%) subjects, lacunae in 36 (48,6,9%) subjects, microbleeds in 28 (37.8%) subjects, and enlarged perivascular spaces in 74 (100%) subjects.

### 4.2. Association between DTI Values and Cognitive Impairment

The mean FA, MD, AD, and RD values for different ROIs in patients with CSVD and their comparison with the controls are presented in Table 2 and Table 3.

The Benjamini–Hochberg method was used to analyze the multiplicity of comparisons, according to which statistically significant differences arose at *p* < 0.022.

Patients with CSVD and the controls had statistically significant differences in their FA, in the anterior frontal dNAWM, posterior frontal pvNAWM, temporoparietal jcNAWM, left anterior and middle CB, anterior, mid-anterior, and mid-posterior CC; MD, in all jcNAWW, anterior and posterior frontal dNAWM, posterior frontal pvNAWM, left anterior and middle CB and right anterior CB, and all parts of CC (Table 2); AD, in the anterior and posterior frontal NAWM, temporoparietal pvNAWM, and mid-posterior CC; and RD, in the anterior frontal and temporoparietal jcNAWM, anterior and posterior frontal dNAWM, posterior frontal pvNAWM, anterior, and middle parts of CB, and anterior, mid-anterior, and mid-posterior CC (Table 3).

Binary logistic regression was used to assess the predictive ability of DTI parameters for the diagnosis of CI in patients with CSVD, where 0 was taken as a binary outcome for SCI and 1 indicated MCI and dementia. All obtained DTI data were included in the logistic regression, which subsequently created an equation featuring the most statistically significant predictor indicators.

The resulting logistic regression model is described in the third step of the step-by-step algorithm for including the predictors. The omnibus tests for the model coefficients show the model’s high statistical significance p=0.00006 (test statistics = 22.120 - χ2 test). The -2log likelihood value was 31.919 in the third step. The R^2^ coefficient of determination was 57.7% of the variance of the dependent variable, which was explained by factors included in the model. According to the Hosmer–Lemeshow goodness-of-fit test, using a predictive model based on these three predictors makes it possible to successfully classify cases as part of dividing the sample into 10 levels (*p* = 0.543). 

Table 4 shows the data of the created model for CI predictors in CSVD. 

In accordance with the model of binary logistic regression, the AD of the posterior frontal pvNAWM (OR 4.050, 95% CI 1.375–11.928), right middle CB (OR 2.966, 95% CI 1.128–7.801), and mid-posterior CC (OR 4.955, 95% CI 1.724–14.242) had the greatest predictive ability.

Following from the model of binary logistic regression, the predicted probability of CI in patients with CSVD (*P*) was calculated using the equation *P* = $\frac{1}{1+e^{-z}}$, where *e* is the base of the natural logarithm and *z* is a linear function and equals *constant + B*_1_ × *χ*_1_ + *B*_2_ × *χ*_2_ + *B*_3_ × *χ*_3_, where *χ*_1,2,3_ are the values of the AD of the posterior frontal pvNAWM, right middle CB, and mid-posterior CC, respectively, while *B*_1,2,3_ are the coefficients of these predictors. 

**Figure 2:**
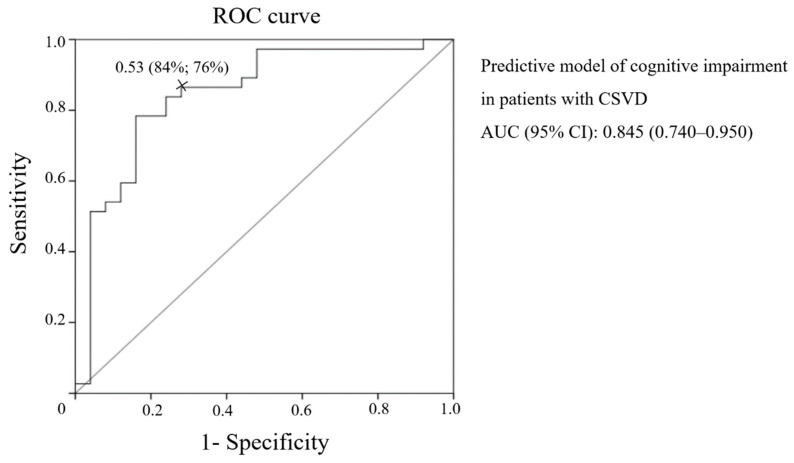
Receiver operating characteristic (ROC) curve of the predictive model of CI in patients with CSVD.

In line with the ROC analysis, the presented model offered good predictive ability for the CI in patients with CSVD (AUC (95% CI): 0.845 (0.740–0.950)) (Figure 2).

According to the ROC analysis the most cutoff value of this predictive model for CI in patients with CSVD was 0.53, where the sensitivity was 84% and the specificity 76%. 

The validity of the AD determinants of CI in patients with CSVD, selected by logistic regression, was confirmed by the significant correlations of these determinants with the MoCA score (for the AD of the posterior frontal pvNAWM r = −0.508, *p* < 0.001; AD of the mid-posterior CC r = −0.559, *p* < 0.001; AD of the right middle CB r = −0.218, *p* = 0.038) and the correspondence of the results when the equation was solved using the patient and control values obtained during neuropsychological testing. 

### 4.3. Association between DTI Parameters and Blood Flow, CSF Flow, and Brain Atrophy

To clarify the values of the leading conditions in ROI data lesions, the main MRI parameters of blood flow, CSF flow, and atrophy in patients and the controls were evaluated (Table 5), as well as their relationships with the AD predictors of CI (Table 6). 

Statistically significant differences between patients with CSVD and the controls were revealed in their sssVBF, aqCSF flow, tBV, vLV, tCSF, tGM, tWM/tBV, and tGM/tBV (Table 5).

All AD predictors had a direct relationship with tCSF. The AD of the posterior frontal pvNAWM and the mid-posterior CC presented a statistically significant direct relationship with arterial Pi, vWMH, and vLV and an inverse relationship with tABF, stVBF, sssVBF, and tGM. The AD of the posterior frontal pvNAWM also had an inverse relationship with tGM/tBV. The AD of the posterior frontal pvNAWM and the right middle CB had a direct relationship with aqCSF flow and sAq (Table 6).

## 5. Discussion

The insertion of DTI ROI analysis data (FA, MD, AD, RD) in the construction of the binary logistic model has made it possible to determine DTI predictors of CI in the patients with CSVD - AD of the pvNAWM, right middle CB and mid-posterior CC. The correlation of these predictors with the severity of WMH, signs of atrophy and also disturbances of blood and CSF flow, all of which are the leading mechanisms of CSVD development, confirms the priority of damage of these zones for the development of CI in the patients with CSVD.

A more time-consuming ROI analysis of the DTI data from patients with CSVD and CI was chosen due to the significant interindividual variability in ventricular size and brain matter when searching for the most sensitive and specific predictors of CI. A large number of ROIs was selected due to the regional selectivity and heterogeneity of the mechanisms of brain damage [20,29,30] and the complexity of the neuropsychological profile [25,31,32,33]. When assessing the severity of CI, we considered a patient’s independence in daily life [38], as well as the results of the MoCA scale [37,51], which showed a correlation with the DTI data in CSVD [17]. The ROIs in the hemispheres were selected according to white matter division in the elderly [36], which most closely matches the blood supply and reflects the difficulty in clarifying the importance of a lesion in the deep or periventricular white matter in CSVD [14,52]. The ROIs were selected in jcNAWM, dNAWM, and pvNAWM along the axes of the anterior and posterior horns, as well as the bodies of the lateral ventricles, since these regions have a large amount of WMH at an advanced stage [10]. Selection of the other ROIs most closely related to CI was carried out in accordance with their recognized divisions [36,48]. According to the binary logistic regression model, the AD values in the posterior frontal pvNAWM, right middle CB, and mid-posterior CC are predictors of CI in CSVD. The correlations between the AD values in these ROIs and the results of the MoCA scale, as well as the solution of the obtained equation using values from patients with CI (MCI and dementia) and subjective cognitive complaints, confirmed the model’s ability to determine the presence of CI.

All the obtained DTI predictors of CI are related to AD and are characterized by elevated values compared to the controls. In a study by Lawrence et al. (2013), a unidirectional increase in AD and RD was found in patients with MCI due to CSVD compared to the controls. At the same time, RD was a stronger predictor of dysregulation, which demonstrated its importance in the development of ischemic demyelination, rather than axonal degeneration [25]. There is reason to believe that an increase in AD may be due to a reduction in axonal density and widening of interstitial spaces, which is typical for the chronic stage. Histopathological studies have previously shown that patients with subcortical progressive vascular encephalopathy have nearly 25% fewer nerve fibers in the CC and frontal white matter [53]. In addition, a reduction in axonal density is associated with an increase in the diameter of the remaining axons, which shows a link with CI severity in patients with multiple sclerosis [54] and possibly alters diffusion in the form of AD elevation. This hypothesis could be supported by the fact that in an experimental cuprizone mouse model, AD decreased at the onset but not in chronic demyelination, despite the presence of axonal degeneration [55]. According to P. J. Winklewski et al. (2018), a significant loss of axons in the chronic stage may lead to a simultaneous increase in AD and RD due to an increase in isotropic diffusion [24]. The experimental and histological data, along with our own suppositions, suggest that this a logical explanation for AD elevation as an equivalent of axonal damage during the chronic stage.

Preserving the structures with the selected ROI predictors is a priority to ensure cognitive functions, as their damage is associated with the development of CI [56,57,58,59]. We postulate that including only three AD predictors in the predictive model of CI in CSVD means that a certain degree of axonal death in these structures is necessary for the development of CI in CSVD. This is consistent with the previously mentioned histological data describing a decrease in the number of axons, predominantly in the CC and frontal lobe white matter, during the advanced stages of the disease [52].

We noted that the ROI predictors were located in anatomical proximity to each other and the floor of the lateral ventricle. Neurofibrillary tangles and breakdown of the ventricular lining were established by Shim et al. (2015) as neuropathological predictors of damage to the periventricular WMH as opposed to deep WMH [34]. This suggests that the damage mechanism in the selected ROI CI predictors involves transependymal CSF transudation followed by axonal damage. The significance of this mechanism in the development of CSVD and the formation of WMH is supported by many researchers [34,60,61,62]. Most likely, CSF transudation triggers the process of axonal damage, similar to the well-studied effect in brain edema, inducing a continuum of anoxic and hypoxic brain injury and the oncotic cell death of myelinated axons, oligodendrocytes, and astrocytes [63,64]. CSF transudation could be a consequence of an increase in CSF pressure, including intraventricular pressure, during certain phases of the cardiac cycle in patients with CSVD. We previously established, in the same group of patients, an association between CI and elevated arterial Pi, thereby demonstrating reduced compliancy and aqCSF flow, which points to compensatory CSF movement [65]. The established link between these indicators and WMH, reduced blood flow in the arteries and veins, and between aqCSF flow and sAq and vLV demonstrate that there is interdependence in the changes between blood flow and CSF flow. Under the conditions of reduced arterial pulse volume and venous hypertension, movement of the CSF is impaired, leading to an increase in CSF pressure during certain phases of the cardiac cycle in relation to the peak arterial load, which may influence the formation of atrophy and WMH [65]. To clarify the importance of this mechanism in damaging the selected ROIs, AD predictors were correlated with the blood flow and CSF flow parameters, as well as various atrophy parameters.

Direct correlations were found between the AD of the posterior frontal pvNAWM and the mid posterior CC with arterial Pi and vWMH, and inverse correlations were found between tABF, stVBF, and sssVBF, while the AD of the posterior frontal pvNAWM and right middle CB had a direct correlation with aqCSF flow. This finding confirms the previously established conditions of blood flow and CSF flow imbalance in the development of CI in patients with CSVD. The role of this mechanism in the development of axonal degeneration can be confirmed by the relationship between the AD of the selected ROIs and vLV and sAq, rather than the volume of the whole brain, its white mattet, and the normalized WM/TBV coefficients. The AD elevation in the posterior frontal pvNAWM and mid posterior CC had a direct correlation with the volume of gray matter and its normalized GM/TBV coefficient which, according to the supposed mechanisms, may also be due to neuronal death caused by an increase in CSF pressure and cortical edema, as a result of the gray matter’s sensitivity [63,64]. These suppositions are indirectly supported by the identified relationship between the AD of all three selected ROIs and the CSF volume and echo the results of studies on the special role of disturbances in CSF circulation in the development of CI and degeneration [66,67]. These results match the conclusions of other studies on the significance of damage to the CC and the periventricular white matter by mechanisms other than deep white matter disease [56,62,68], as well as the links between periventricular WMH and degenerative processes, such as increased amyloid [69] and severe neurofibrillary tangles [34].

Therefore, binary logistic regression using the FA, MD, AD, and RD in different ROIs of patients with MRI signs of CSVD established that the predictors of CI are AD of the posterior frontal pvNAWM, right middle CB, and mid-posterior CC. The obtained data correspond to the significance of axonal damage in these regions and the mechanisms that determine the formation of such damage as a mandatory condition for the development of CI in CSVD. The direct relationship between AD predictors and arterial Pi and aqCSF flow and the inverse relationship with tABF, stVBF, and sssVBF indicate the significance of the disturbances in the interactions between blood flow and CSF flow in the development of CI in patients with CSVD. The locations of the AD predictors near each other and the floor of the lateral ventricle and their direct relationship with tCSF, tWMH, vLV, and sAq, rather than tBV, tWM, and WM/TBV, indicate that axonal degeneration in the ROI data is most likely related to CSF transudation. This may be due to, for example, an increase in CSF pressure during certain phases of the cardiac cycle under the conditions of a decrease in arterial compliance and venous sinuses hypertension. The established predictors of CI and the supposed mechanisms of their formation can help clarify the interactions between vascular and degenerative pathologies and potentially develop methods to prevent the associated CI.

## Figures and Tables

**Table 1:** Characteristics of patients with cerebral small vessel disease (CSVD) and the controls.

Parameters	CSVD (*n* = 74)	Control (*n* = 18)	*p*
Sex, women (*n*, %)	48 (64.8%)	12 (66.6%)	0.559
Age, years (mean ± SD, min., max.)	60.7 ± 6.9, min. 45, max. 70	57.8 ± 5.9, min. 45, max. 67	0.084
Education, years (mean ± SD, min., max.)	14.3 ± 2.4, min. 8, max. 20	15.7 ± 2.2, min. 11, max. 20	0.136
AH (*n*, %)	60 (81.1%)	7 (38.9%)	**<0.001**
Degree of AH (*n*, %)			
stage 1	7 (9.5%)	4 (22.2%)	
stage 2	12 (16.2%)	2 (11.1%)	
stage 3	41 (55.4%)	1 (5.6%)	
DM type 2 (*n*, %)	16 (21.6%)	0 (0%)	0.065
Hypercholesterolemia (total cholesterol > 6.2 mmol/L or *statin use)* (*n*, %)	34 (45.9%)	6 (33.3%)	0.128
Smoking (*n*, %)	21 (28.4%)	6 (33.3%)	0.559
Obesity (body mass index > 30 kg/m²) (*n*, %)	27 (36.5%)	1 (5.6%)	0.354
MoCA score (Me [Q25%; Q75%])	25 [22; 27]	29 [28; 30]	**<0.001**
Cognitive impairment (*n*, %):	74 (100%)		
SCI	29 (39.2%)		
MCI	33 (44.6%)		
dementia	12 (16.2%)		
WMH, Fazekas Scale (*n*, %)	74 (100%)		
grade 1	19 (25.6%)		
grade 2	23 (31.1%)		
grade 3	32 (43.3%)		
Lacunae (*n*, %)	36 (48.6%)		
Microbleeds (*n*, %)	28 (37.8%)		
Perivascular spaces (*n*, %)	74 (100%)		

Abbreviations and notes: values in bold are statically significant differences. AH—arterial hypertension, DM—diabetes mellitus, SCI—subjective cognitive impairment, MCI— mild cognitive impairment, WMH—white matter hyperintensity.

**Table 2:** Fractional anisotropy (FA) and mean diffusivity (MD) for different ROIs in the subjects with CSVD and the controls.

ROI	FA (Mean ± SD)		*p*	MD (Mean ± SD)		*p*
	CSVD (*n* = 74)	Control (*n* = 18)		CSVD (*n* = 74)	Control (*n* = 18)	
Anterior frontal						
jcNAWM	33.14 ± 7.99	29.03 ± 7.74	0.023	9.26 ± 0.73	10.29 ± 0.97	**<0.001**
dNAWM	33.29 ± 10.04	28.55 ± 6.97	**0.014**	9.12 ± 0.82	10.05 ± 1.16	**<0.001**
pvNAWM	27.15 ± 8.94	34.15 ± 11.49	0.047	9.32 ± 0.82	10.09 ± 1.44	0.041
Posterior frontal						
jcNAWM	35.86 ± 7.52	35.89 ± 9.28	0.919	9.50 ± 0.75	10.18 ± 1.16	**0.015**
dNAWM	34.29 ± 7.10	34.16 ± 8.16	0.646	8.67 ± 0.78	9.69 ± 1.09	**<0.001**
pvNAWM	36.80 ± 9.78	27.67 ± 9.38	**0.004**	8.64 ± 0.80	11.45 ± 2.34	**<0.001**
Temporoparietal						
jcNAWM	43.84 ± 7.78	35.26 ± 10.35	**0.002**	8.58 ± 0.54	9.78 ± 1.25	**<0.001**
dNAWM	34.37 ± 7.22	35.28 ± 10.83	0.935	9.43 ± 1.14	9.67 ± 1.21	0.584
pvNAWM	47.74 ± 7.89	50.69 ± 12.67	0.256	9.38 ± 0.52	10.19 ± 1.38	**0.022**
Anterior CB						
left	46.69 ± 8.38	39.47 ± 10.42	**0.017**	9.41 ± 1.17	10.25 ± 1.13	**0.019**
right	46.32 ± 9.11	41.06 ± 11.89	0.059	8.69 ± 1.10	9.86 ± 1.28	**0.001**
Middle CB						
left	58.86 ± 10.96	49.12 ± 12.41	**0.005**	8.46 ± 0.88	9.29 ± 1.17	**0.002**
right	54.57 ± 11.89	49.28 ± 11.42	0.068	8.55 ± 0.97	9.09 ± 1.42	0.106
Posterior CB						
left	35.81 ± 11.77	33.79 ±1 3.61	0.370	10.22 ± 4.34	9.62 ± 1.25	0.306
right	37.03 ± 18.91	33.30 ± 12.46	0.466	9.19 ± 1.08	9.65 ± 1.67	0.121
Anterior CC	90.93 ± 4.82	76.83 ± 14.81	**<0.001**	7.48 ± 0.76	9.41 ± 2.52	**0.002**
Mid-anterior CC	61.48 ± 7.92	46.35 ± 14.77	**<0.001**	11.36 ± 1.69	13.65 ± 3.17	**0.004**
Mid-posterior CC	57.61 ± 13.49	48.25 ± 15.21	**0.040**	12.16 ± 1.98	14.99 ± 3.90	**0.022**
Posterior CC	86.99 ± 4.13	79.35 ± 12.54	0.018	8.53 ± 1.04	9.79 ± 2.23	**0.004**
Hippocampus						
left	30.51 ± 9.45	30.68 ± 8.69	0.946	10.82 ± 1.50	10.88 ± 1.77	0.899
right	31.34 ± 7.16	30.09 ± 9.59	0.858	10.29 ± 1.05	10.67 ± 1.91	0.273

Abbreviations and notes: values in bold are statically significant differences. FA—fractional anisotropy (×10^−2^); MD—mean diffusivity (mm^2^/s × 10^−4^); jcNAWM—juxtacortical normal appearing white matter, dNAWM—deep normal appearing white matter, pvNAWM—periventricular normal appearing white matter, CB—cingulum bundle, CC—corpus callosum.

**Table 3:** AD and radial diffusivity (RD) for different ROIs in subjects with CSVD and the controls.

ROI	AD (Mean ± SD)		*p*	RD (Mean ± SD)		*p*
	CSVD (*n* = 74)	Control (*n* = 18)		CSVD (*n* = 74)	Control (*n* = 18)	
**Anterior frontal**						
**jcNAWM**	12.52 ± 1.14	13.38 ± 1.29	**<0.001**	7.62 ± 0.91	8.75 ± 1.17	**0.017**
**dNAWM**	12.19 ± 1.13	12.99 ± 1.26	**0.001**	7.58 ± 0.94	8.59 ± 1.01	**0.008**
**pvNAWM**	11.94 ± 0.92	13.87 ± 2.18	**0.001**	8.01 ± 1.09	8.20 ± 1.66	0.582
**Posterior frontal**						
**jcNAWM**	13.19 ± 1.20	14.06 ± 1.59	0.064	7.65 ± 0.85	8.24 ± 1.32	0.026
**dNAWM**	11.83 ± 1.03	13.21 ± 1.55	**0.005**	7.09 ± 0.88	7.94 ± 1.19	**0.002**
**pvNAWM**	12.39 ± 0.82	14.71 ± 2.34	**<0.001**	6.76 ± 1.22	9.82 ± 2.53	**<0.001**
**Temporoparietal**						
**jcNAWM**	12.77 ± 1.21	13.48 ± 1.48	**<0.001**	6.48 ± 0.69	7.93 ± 1.55	0.053
**dNAWM**	12.98 ± 1.45	13.35 ± 1.57	0.754	7.66 ± 1.16	7.84 ± 1.47	0.618
**pvNAWM**	14.45 ± 1.72	16.45 ± 2.61	0.730	6.85 ± 0.76	7.05 ± 1.68	0.636
Anterior CB						
left	14.57 ± 1.59	14.79 ± 1.74	**0.010**	6.83 ± 1.49	7.98 ± 1.35	0.798
right	13.45 ± 1.65	14.45 ± 1.96	**0.002**	6.31 ± 1.20	7.56 ± 1.63	**0.001**
Middle CB						
left	14.46 ± 2.52	14.78 ± 2.29	**0.005**	5.46 ± 1.15	6.54 ± 1.41	0.630
right	14.39 ± 2.19	14.45 ± 2.63	0.917	5.63 ± 1.13	6.40 ± 1.41	0.976
Posterior CB						
left	14.03 ± 4.35	13.38 ± 2.25	0.545	8.31 ± 4.53	7.74 ± 1.59	0.608
right	13.05 ± 2.60	13.39 ± 2.31	0.201	7.26 ± 1.88	7.78 ± 1.57	0.290
Anterior CC	19.26 ± 1.79	19.85 ± 2.43	0.259	1.62 ± 0.77	4.19 ± 3.09	0.454
Mid-anterior CC	20.41 ± 2.46	20.94 ± 2.29	0.407	6.83 ± 1.61	9.99 ± 3.86	0.355
Mid-posterior CC	20.91 ± 2.24	23.36 ± 2.89	**<0.001**	7.79 ± 2.49	10.81 ± 4.67	**0.010**
Posterior CC	20.34 ± 1.81	21.26 ± 2.21	0.075	2.62 ± 1.37	4.05 ± 2.68	0.098
Hippocampus						
left	14.35 ± 1.31	14.51 ± 1.85	0.678	9.06 ± 1.84	9.06 ± 2.01	0.976
right	13.93 ± 1.51	14.15 ± 1.98	0.609	8.48 ± 1.07	8.92 ± 2.12	0.393

Abbreviations and notes: values in bold are statically significant differences. AD—axial diffusivity (mm^2^/s × 10^−4^); RD—radial diffusivity (mm^2^/s × 10^−4^); jcNAWM—juxtacortical normal appearing white matter, dNAWM—deep normal appearing white matter, pvNAWM—periventricular normal appearing white matter, CB—cingulum bundle, CC—corpus callosum.

**Table 4:** Predictors of cognitive impairment (CI) severity (binary logistic regression, *p* < 0.001).

Predictors	B (Coefficients of Predictors)	*p*	OR	95% CI	
				Lower	Upper
AD of posterior frontal pvNAWM (*χ*_1_)	11,053.52	0.014	4.050	1.375	11.928
AD of right middle CB (*χ*_2_)	7248.06	0.011	2.966	1.128	7.801
AD of mid-posterior CC (*χ*_3_)	6310.07	0.046	4.955	1.724	14.242
Constant	–39.81	0.025			

**Table 5:** MRI results in subjects with CSVD and the controls.

Parameters	CSVD (*n* = 74)	Control (*n* = 18)	*p*
tABF (ml/min)	506.86 ± 128.25	566.22 ± 127.84	0.159
stVBF (ml/min)	86.15 ± 23.29	99.39 ± 18.93	0.067
sssVBF (ml/min)	241.85 ± 59.95	285.94 ± 62.21	**0.024**
Pi	1.12 ± 0.29	1.05 ± 0.23	0.351
aqCSF flow (mm^3^/s)	74.16 ± 65.97	47.76 ± 19.46	**0.011**
sAq (mm^2^)	8.18 ± 3.28	6.47 ± 1.09	0.093
tBV (cm^3^)	1009.91 ± 113.57	1102.74 ± 68.59	**0.004**
vLV (cm^3^)	39.92 ± 24.64	19.79 ± 9.42	**0.001**
tCSF (cm^3^)	497.01 ± 112.93	390.32 ± 82.15	**0.001**
tWM (cm^3^)	450.30 ± 59.79	465.86 ± 43.43	0.276
tGM (cm^3^)	559.61 ± 74.09	636.88 ± 43.19	**0.001**
tWM/tBV	0.446 ± 0.037	0.422 ± 0.024	**0.009**
tGM/tBV	0.554 ± 0.037	0.577 ± 0.024	**0.009**
vWMH (cm^3^)	22.963 ± 13.6		

Abbreviations and notes: values in bold are statically significant differences; numbers represent means ± SD. Abbreviations: tABF—total arterial blood flow; stVBF—straight sinus venous blood flow; sssVBF—superior sagittal sinus venous blood flow; Pi—arterial pulsatility index; aqCSF flow— aqueduct cerebrospinal fluid flow; sAq—surface area aqueduct; tBV—total brain volume; vLV—volume of lateral ventricles; tCSF—total cerebrospinal fluid; tWM—total white matter; tGM—total gray matter; tWM/tBV—total white matter/total brain volume; tGM/tBV—total gray matter/total brain volume; vWMH—volume of white matter hyperintensity.

**Table 6:** Relationships between the diffusion tensor imaging (DTI) predictors of CI and the phase contrast MRI (PC–MRI) and morphometry values.

Parameters	AD of Posterior Frontal pvNAWM	AD of Mid−Posterior CC	AD of Right Middle CB
tABF	−0.451 **	−0.406 **	0.152
stVBF	−0.461 **	−0.371 **	0.222
sssVBF	−0.317 **	−0.415 **	0.218
Pi	0.313 *	0.406 **	0.030
aqCSF flow	0.269 *	0.073	0.234 *
sAq	0.237 *	0.200	0.328 **
tBV	−0.189	−0.167	0.020
vLV	0.580 **	0.377 **	0.135
tCSF	0.570 **	0.308 **	0.221 *
tWM	0.013	−0.026	0.089
tGM	−0.294 *	−0.230 *	−0.038
tWM/tBV	0.285	0.173	0.132
tGM/tBV	−0.285 *	−0.173	−0.132
vWMH	0.410 **	0.360 **	0.080

Abbreviations and notes: values in bold are statically significant differences; numbers represent the correlation coefficients, * *p* < 0.05, ** *p* < 0.001. Abbreviations: tABF—total arterial blood flow, stVBF—straight sinus venous blood flow, sssVBF—superior sagittal sinus venous blood flow, Pi—pulsatility index, aqCSF flow—aqueduct cerebrospinal fluid flow, sAq—surface area aqueduct, tBV—total brain volume, vLV—volume of lateral ventricles, tCSF—total cerebrospinal fluid, tWM—total white matter, tGM—total gray matter, tWM/tBV—total white matter/ total brain volume, tGM/tBV—total gray matter /total brain volume, vWMH—volume of white matter hyperintensity.

## Abbreviations

AD	Axial diffusivity
AH	Arterial hypertension
aqCSF flow	Aqueduct cerebrospinal fluid flow
AUC	Area under the curve
CB	Cingulum bundle
CC	Corpus callosum
CI	Confidence interval
CSF	Cerebrospinal fluid
CSVD	Cerebral small vessel disease
DM	Diabetes mellitus
dNAWM	Deep normal appearing white matter
DTI	Diffusion tensor imaging
FA	Fractional anisotropy
jcNAWM	Juxtacortical normal appearing white matter
MCI	Mild cognitive impairment
MD	Mean Diffusivity
MoCA	Montreal Cognitive Assessment
MRI	Magnetic resonance imaging
NAWM	Normal appearing white matter
OR	Odds ratio
PC-MRI	Phase contrast magnetic resonance imaging
Pi	Pulsatility index
pvNAWM	Periventricular normal appearing white matter
RD	Radial diffusivity
ROC	Receiver operating characteristic
ROI	Region of interest
stVBF	Straight sinus venous blood flow
SCI	Subjective cognitive impairment
sssVBF	Superior sagittal sinus venous blood flow
sAq	Surface area aqueduct
tABF	Total arterial blood flow
tBV	Total brain volume
tCSF	Total cerebrospinal fluid
tGM	Total gray matter
tWM	Total white matter
vLV	Volume of lateral ventricles
vWMH	Volume of white matter hyperintensity
WMHs	White matter hyperintensities
